# The *WIPI* Gene Family and Neurodegenerative Diseases: Insights From Yeast and *Dictyostelium* Models

**DOI:** 10.3389/fcell.2021.737071

**Published:** 2021-09-01

**Authors:** Olivier Vincent, Laura Antón-Esteban, Miranda Bueno-Arribas, Alba Tornero-Écija, María-Ángeles Navas, Ricardo Escalante

**Affiliations:** ^1^Instituto de Investigaciones Biomédicas Alberto Sols CSIC/UAM, Madrid, Spain; ^2^Departamento de Bioquímica y Biología Molecular, Facultad de Medicina, Universidad Complutense de Madrid, Madrid, Spain

**Keywords:** autophagy, BPAN, *Dictyostelium discoideum*, *Saccharomyces cerevisiae*, PROPPIN, Vmp1, WDR45, *WIPI*

## Abstract

WIPIs are a conserved family of proteins with a characteristic 7-bladed β-propeller structure. They play a prominent role in autophagy, but also in other membrane trafficking processes. Mutations in human *WIPI4* cause several neurodegenerative diseases. One of them is BPAN, a rare disease characterized by developmental delay, motor disorders, and seizures. Autophagy dysfunction is thought to play an important role in this disease but the precise pathological consequences of the mutations are not well established. The use of simple models such as the yeast *Saccharomyces cerevisiae* and the social amoeba *Dictyostelium discoideum* provides valuable information on the molecular and cellular function of these proteins, but also sheds light on possible pathways that may be relevant in the search for potential therapies. Here, we review the function of WIPIs as well as disease-causing mutations with a special focus on the information provided by these simple models.

## Introduction

**FIGURE 1:**
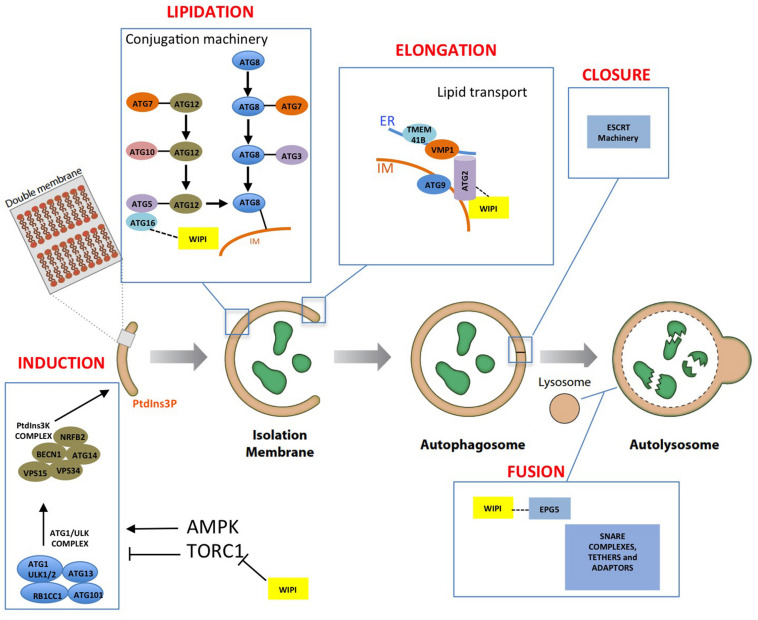
Autophagosome formation and the autophagic machinery. The different steps of autophagosome formation are shown together with the proteins and protein complexes involved in autophagy regulation. WIPIs (yellow boxes) are involved in different steps of autophagosome formation (see the text for a detailed explanation of the specific WIPIs involved in each step).

Degradation and recycling of cellular components are essential for cell homeostasis. Autophagy (from the Greek, “self-eating”) includes several pathways that deliver different types of cargos to lysosomes for degradation. Macroautophagy (referred to as autophagy hereafter) is characterized by the formation of double-membrane vesicles, known as autophagosomes, which can engulf non-specific cellular material (bulk autophagy) or specific cargoes, such as protein aggregates or defective organelles (selective autophagy). After the fusion of autophagosomes with the lysosomes, the simple biochemical compounds generated during the degradation process are transported to the cytosol and reused for energy production or recycling (see recent reviews Lahiri et al., 2019; Melia et al., 2020; Gubas and Dikic, 2021). Autophagosome biogenesis is a highly regulated process involving different stages: induction, lipidation, and elongation of the autophagosome membrane (also known as the isolation membrane or phagophore), vesicle closure, and fusion with lysosomes. These steps are controlled by protein complexes formed mainly by the so-called Atg proteins. Figure 1 summarizes schematically the essential components of the autophagy machinery and their function in the different steps of autophagosome formation.

The nutritional and energetic status of cells regulates autophagy through two master regulators, TORC1 and AMPK (Hindupur et al., 2015). These kinases oppositely control autophagy through phosphorylation of key subunits of two essential complexes, Atg1/ULK and PI3KC3. Atg1/ULK kinase complex composed of Atg1, Atg13, Atg17, Atg29, and Atg31 in *Saccharomyces cerevisiae* (yeast hereafter), and by ULK1/2, ATG13, ATG101, and FIP200 in mammalian cells (Hurley and Young, 2017). Atg1, Atg13, and Atg101 have also been identified in *Dictyostelium discoideum* (Otto et al., 2004; Mesquita et al., 2015). During nucleation the Atg1/ULK complex is activated and recruited to specialized regions of the endoplasmic reticulum (ER) through interaction with VAMP-associated proteins (VAPs) (Zhao et al., 2018). The Atg1/ULK complex subsequently activates the PI3KC3 complex composed of Vps34, Vps15, Atg6, and Atg14 in yeast and VPS34, VPS15, BECN1, NRFB2, and ATG14 in mammals (Hurley and Young, 2017). The homologues of all these components can also be recognized in *Dictyostelium* (Calvo-Garrido et al., 2010; Fischer and Eichinger, 2019). The phosphoinositide 3-kinase Vps34/VPS34 generates PtdIns3P by phosphorylation of phosphatidylinositol in the nascent autophagosome membrane and in the specialized ER-derived structure known as omegasome (Axe et al., 2008; Nishimura et al., 2017). The incorporation of Atg9 containing vesicles, which seed the initial autophagosome membrane, is also under the control of the Atg1/ULK complex (Papinski et al., 2014; Karanasios et al., 2016; Nishimura et al., 2017; Sawa-Makarska et al., 2020).

The PtdIns3P generated at the autophagosome assembly site recruits a family of proteins called WIPIs (WD-repeat protein Interacting with phosphoinositides), also known as PROPPINs (beta-propellers that bind polyphosphoinositides), which are necessary for the subsequent recruitment of other autophagic proteins. Their structure, function and role in disease are the focus of this review. One of the proteins recruited by WIPIs is Atg2 in yeast (ATG2A/ATG2B in mammalian cells), a lipid transport protein that appears to be responsible for autophagosome membrane elongation through lipid transport from the ER (Maeda et al., 2019; Osawa et al., 2019, 2020; Valverde et al., 2019). Autophagosome membrane elongation also requires the function of several transmembrane proteins, ATG9 in the phagophore membrane, and VMP1 and TMEM41B in the ER membrane, which have recently been reported to have scramblase activity *in vitro* (Valverde et al., 2019; Matoba et al., 2020; Li et al., 2021). Since lipids are extracted by Atg2 from the outer leaflet of the ER membrane and delivered to the outer leaflet of the autophagosome membrane, lipid transport from one leaflet to the other that is accomplished by these scramblases is necessary to equilibrate the lipid composition on both sides of the bilayer of the donor and acceptor membranes.

Atg8 lipidation, a ubiquitin-like mechanism that covalently attaches the protein Atg8 to the autophagosome membrane, is also required for elongation and closure of the autophagosome membrane. In mammalian cells, Atg8 proteins are classified into three groups: LC3, GABARAP, and GABARAPL (Slobodkin and Elazar, 2013). In this process, the Atg12 protein is first conjugated to Atg5 and this complex interacts non-covalently with Atg16. The WIPI-mediated recruitment of the Atg12-Atg5/Atg16 complex to the autophagosome membrane promotes the conjugation of Atg8 to phosphatidyl-ethanolamine (PE) in a process that also involves the E1-like and E2-like enzymes Atg7 and Atg3 (Geng and Klionsky, 2008). The proteins involved in this ubiquitin-like mechanism are highly conserved in all eukaryotes, including *Dictyostelium* (Calvo-Garrido et al., 2010; Mesquita et al., 2017; Fischer and Eichinger, 2019). Closure of the autophagosome membrane and fusion with lysosomes requires the function of the ESCRT machinery and SNARE complexes, respectively (Lőrincz and Juhász, 2019).

Autophagy influences virtually all cellular processes, as it regulates the cellular response to the immediate metabolic demands and also plays a direct role in the quality control of protein and organelles. Consequently, autophagy has a major impact at the organismal level and regulates many physiological functions in mammalian cells, including differentiation and development, tissue and organ homeostasis, immunity and aging. Mutations in autophagy-related genes have been associated with different diseases (see a recent review for an overview; Mizushima and Levine, 2020). Mutations in *WIPI* genes lead to severe neurological disorders, including neurodegeneration, intellectual disability, epilepsy, developmental abnormalities, etc. Specifically, mutations in the *WIPI4/WDR45* gene lead to BPAN (beta propeller-associated neurodegeneration), an ultra-rare disease with no cure that affects children. The use of simple model organisms such as Yeast and *Dictyostelium* can help to understand the molecular basis of these diseases.

## The Yeast and *Dictyostelium* Models in Autophagy and Disease

Yeast has been the pioneer model in which most of the components of the autophagic machinery have been discovered and characterized. The lack of autophagy in this organism leads to reduced survival under conditions of nitrogen starvation. Nobel laureate Yoshinory Ohsumi took advantage of the powerful genetics and manipulability of yeast to screen for genes required for autophagy, whose encoded proteins were named Atg proteins (Tsukada and Ohsumi, 1993; Scott et al., 1996; Matsuura et al., 1997). Although the simplicity of the yeast model has allowed the fundamental leap of identifying the key components of the autophagic machinery, this model lacks the complexity of phenotypes usually associated with autophagy dysfunction in multicellular organisms. *Dictyostelium* can therefore complement the use of yeast, primarily for certain genes or pathways that are not found in yeast, while conserved between *Dictyostelium* and animal cells.

**FIGURE 2:**
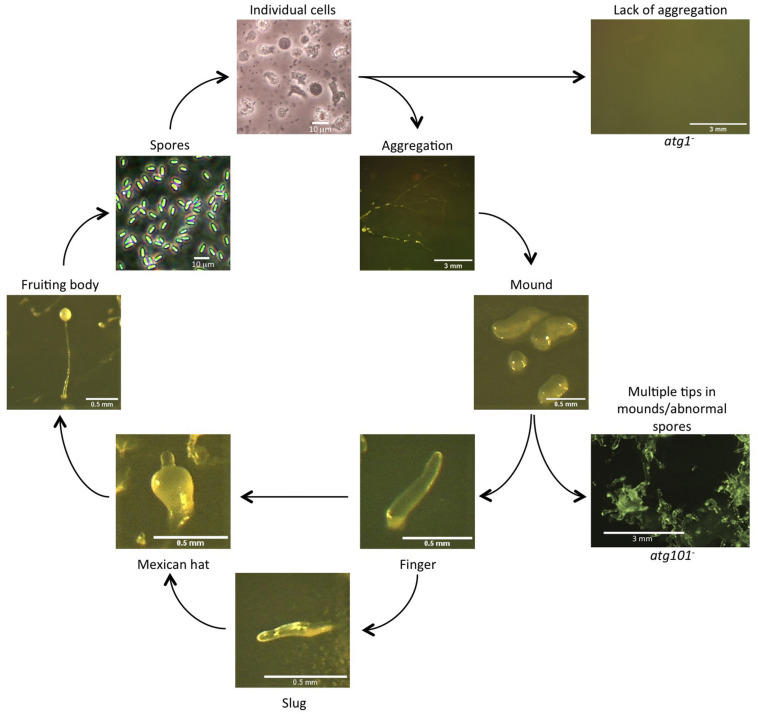
Autophagy is required for *Dictyostelium* development. Representative pictures of the *Dictyostelium* life cycle are shown. Strains defective in autophagy (right panels) show distinct phenotypes, such as lack of aggregation (Atg1 mutant is shown as an example) or abnormal development with a lack of spores (Atg101 mutant is shown as an example). Scale bars are shown in each photograph.

The species *Dictyostelium discoideum* was discovered in 1935 by Kenneth Raper in Western North Carolina (United States) (Raper, 1935), and the potential of this organism as an experimental model became apparent very early on. *Dictyostelium* belongs to the amoebozoa group, and although this group of organisms diverged before the opistokonta (fungi and animals), it retains many features of animal cells that have been lost during the evolution of fungi. Cell motility and chemotaxis, phagocytosis and macropynocytosis are very similar to those observed in animal cells and *Dictyostelium* presents a multicellular stage that allows the study of cell differentiation and morphogenesis (see this series of reviews collected in a special issue dedicated to *Dictyostelium* in *IJDB* (Araki and Saito, 2019; Batsios et al., 2019; Bloomfield, 2019; Bozzaro, 2019; Consalvo et al., 2019; Escalante and Cardenal-Muñoz, 2019; Farinholt et al., 2019; Fey et al., 2019; Fischer and Eichinger, 2019; Ishikawa-Ankerhold and Müller-Taubenberger, 2019; Jaiswal et al., 2019; Kawabe et al., 2019; Kay et al., 2019; Knecht et al., 2019; Kundert and Shaulsky, 2019; Kuspa and Shaulsky, 2019; Medina et al., 2019; Nanjundiah, 2019; Pal et al., 2019; Pearce et al., 2019; Pergolizzi et al., 2019; Schaf et al., 2019; Vines and King, 2019). Individual *Dictyostelium* cells ingest bacteria and yeasts in soil and the transition to a multicellular state, triggered when the food source is depleted, is accomplished by aggregation of preexisting cells. This developmental program culminates in the formation of a fruiting body containing spores (Figure 2). As this process takes place in the absence of nutrients, autophagy is essential to sustain the energy-demanding processes of chemotaxis, morphogenesis and cell differentiation. Therefore, autophagy dysfunction results in distinct phenotypes that vary in severity from the complete absence of development to the formation of aberrant, multi-tipped mounds that may eventually form abnormal fruiting bodies containing non-viable spores (Otto et al., 2003, 2004; Calvo-Garrido and Escalante, 2010; Tung et al., 2010; Mesquita et al., 2015; Muñoz-Braceras et al., 2015; Xiong et al., 2015, 2018; Fischer et al., 2019; Sharma et al., 2019; Yamada and Schaap, 2019, 2020; Karow et al., 2020; Figure 2). *Dictyostelium* has become a useful model for studying autophagy. The function of many autophagy genes have been analyzed in this organism and diverse techniques have been fine-tuned to precisely study this process (Calvo-Garrido et al., 2010; Mesquita et al., 2017; Fischer and Eichinger, 2019). In addition, *Dictyostelium* expresses some key autophagic proteins absent in yeast but present in animal cells. Notable examples are the Atg1/ULK complex protein Atg101 (Mesquita et al., 2015), the ER protein Vmp1 (Calvo-Garrido et al., 2008, 2014; Calvo-Garrido and Escalante, 2010), the Atg16 protein containing a C-terminal WD-repeat domain also present in mammalian ATG16L (Xiong et al., 2018) but absent in the yeast homolog, the autophagy regulators KinkyA and Bcas3 (Yamada and Schaap, 2020), and orthologues of the Gamma-secretase PSEN1 (psenA and psenB), NCSTN (nicastrin), and APH1 (gamma-secretase subunit Aph-1) (Sharma et al., 2019).

The use of simple model organisms, such as yeast and *Dictyostelium*, has allowed fundamental advances to be made in the basic principles of many diseases, contributing to the 3R principle (replacing, reducing and refining the use of animals in research, directive 2010/63/EU). The high conservation of protein and signaling pathways during evolution makes it possible to study diseases at different levels: (i) At the molecular level, for example by analyzing the biochemical function, structure and interactions of proteins involved in the disease; (ii) At the cellular and organism level, by analyzing cell physiology, affected pathways, differentiation and development of diseased models; and (iii) At the pharmacological levels by studying the mechanism of action of drugs or even the search for new therapeutic compounds for well-conserved pathways (see some recent works as examples of point iii; Cocorocchio et al., 2017; Schaf et al., 2019; Perry et al., 2020; Warren et al., 2020).

## The Function of WIPIs in the Different Steps of Autophagosome Formation

**FIGURE 3:**
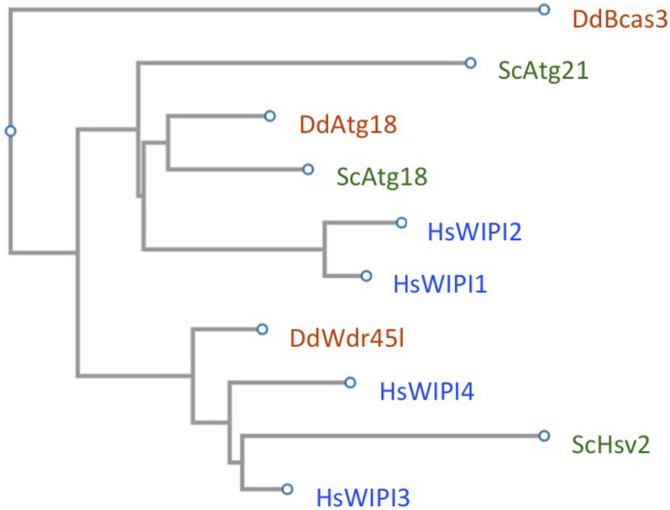
The WIPI family in yeast, *Dictyostelium* and human. The amino acid sequences of yeast Atg18, Atg21, and Hsv2 (UniProt entry: P43601; Q02887; P50079), *Dictyostelium* Atg18 and Wdr45l (UniProt entry: Q54NA2; Q54SA0), and the human WIPI1, WIPI2, WDR45B/WIPI3, and WDR45/WIPI4 (UniProt entry: Q5MNZ9; Q9Y4P8; Q5MNZ6; Q9Y484), were used for a phylogenetic analysis using Fast Tree (https://www.genome.jp/tools-bin/clustalw). The *Dictyostelium* proppin Bcas3 (UniProt entry: Q559F5) was used as an outgroup.

The number of members of this protein family varies among organisms. In mammalian cells there are four members (WIPI1, WIPI2, WDR45B/WIPI3, and WDR45/WIPI4), three in yeast (Atg18, Atg21, and Hsv2) and two in *Dictyostelium* (Atg18 and Wdr45l). A phylogenetic analysis based on sequence similarity clusters WIPI sequences into two groups (Figure 3). One contains the highly similar WIPI1 and WIPI2 along with *Dictyostelium* Atg18 and yeast Atg18 and Atg21. In the other branch, human WIPI3 and WIPI4 are found together with *Dictyostelium* Wdr45l and yeast Hsv2 (Figure 3). Despite the apparent clarity in their distribution, the functions attributed to each member do not always correspond to this position based on sequence similarity. The most striking example is that of Hsv2, which is the closest sequence homologue in yeast of human WIPI3/4 and *Dictyostelium* Wdr45l, proteins that play a prominent role in autophagy, whereas Hsv2 does not appear to play a role in bulk autophagy in yeast (Krick et al., 2008). Next, we will describe in detail and in a comparative way the functions attributed to the human, yeast and *Dictyostelium* WIPI proteins in the different steps of autophagosome formation (Figure 1 shows the WIPIs in their proposed functions throughout the different steps of autophagosome formation).

### Induction Step

The induction of autophagy is regulated by the interplay between AMPK and TORC1. AMPK activates and TORC1 inhibits autophagy. TORC1 is activated by growth factor through RHEB, and aminoacids through RRAG/Rag GTPases that activate TORC1 on the surface of lysosomes, leading to phosphorylation of the ATG1/ULK complex and inhibition of autophagy. AMPK activates autophagy through phosphorylation and activation of the TORC1 inhibitor TSC1/2. In addition, AMPK activates autophagy directly through phosphorylation and inhibition of TORC1, and also through activation by phosphorylation of the ATG1/ULK complex. Analysis of the WIPI3 interactome suggests a regulatory role of this protein in TORC1 inhibition through the TSC1/2 complex. Specifically, WIPI3 interacts with TSC1 when the latter is phosphorylated by AMPK. The complex formed by WIPI3/TSC1/2/FIP200 localizes to the lysosome when TORC1 is absent, suggesting a role in inhibiting and releasing TORC1 from lysosomes to activate autophagy (Bakula et al., 2017). Upon starvation, WIPI3 also translocates to the nascent autophagosome colocalizing with FIP200 and other ATG proteins (Bakula et al., 2017). This possible role of human WIPI3 in the initiation step contrasts with the phenotype associated to human WIPI3 or WIPI4 knock-down (KD) (Bakula et al., 2017) and to Wdr45l knock-out (KO) in *Dictyostelium* (Tornero-Écija et al., 2021), as autophagy is blocked at a later step, following recruitment of the conjugation machinery and lipidation of Atg8/LC3.

### Elongation and Atg8/LC3 Lipidation

WIPI proteins play an essential role in the recruitment of the conjugation machinery responsible for the lipidation of Atg8/LC3 protein at the phagophore membrane. In particular, the interaction of human WIPI2 with ATG16L1 mediates the recruitment of the ATG12-ATG5/ATG16L1 complex (Dooley et al., 2014; Fracchiolla et al., 2020). Accordingly, WIPI2 KD results in a large reduction in LC3 lipidation and autophagosome formation (Polson et al., 2010; Dooley et al., 2014; Bakula et al., 2017). There is also an accumulation of PtdIns3P and DFCP1 (double FYVE-domain containing protein 1) positive structures in WIPI2-depleted HEK293A cells, suggesting a defect in the omegasome structure (Polson et al., 2010).

WIPI1 has been proposed to assist WIPI2 in the recruitment of the conjugation machinery although its role is not essential (Bakula et al., 2017, 2018). Accordingly, WIPI1 KD in G361 cells does not prevent the formation of autophagosomes although a slight decrease in the number of autophagosomal structures is observed (Bakula et al., 2017). However, there is no detectable effect on LC3 lipidation (Proikas-Cezanne et al., 2015), indicating that WIPI1 is partially dispensable for the recruitment of the conjugation machinery.

In yeast, Atg21, like WIPI2 in humans, interacts with Atg16 and is essential for effective recruitment and lipidation of Atg8 (Juris et al., 2015; Krick and Thumm, 2016; Sawa-Makarska et al., 2020). Interestingly, the closest sequence homolog of Atg21 and WIPI2 in *Dictyostelium*, termed Atg18, is largely dispensable for autophagy, as Atg8-containing autophagosomes are formed in Atg18 KO cells, although a slight reduction in autophagy flux can be detected (Tornero-Écija et al., 2021). The other WIPI protein in *Dictyostelium*, Wdr45l, does not appear to be required for lipidation despite the complete block in autophagy, as abnormal Atg8-containing structures are detected in the Wdr45l KO strain (Tornero-Écija et al., 2021). This raises the possibility that in *Dictyostelium*, recruitment of the conjugation machinery is largely independent of WIPI proteins. The existence of alternative mechanisms to WIPIs in this process is consistent with the observation that LC3 lipidation is not completely blocked in mammalian cells depleted of WIPI1 or WIPI2 (Polson et al., 2010), and that a C-terminal region of ATG16L1 can bind to membranes on its own and sustain LC3 lipidation in the absence of WIPI2 (Lystad et al., 2019). Interestingly, this C-terminal extension is absent in yeast Atg16 but present in the *Dictyostelium* Atg16 homolog (Xiong et al., 2018), which could explain the apparently minor role of WIPIs in the conjugation process in this organism, although no direct study has been performed on the ability of this protein region in *Dictyostelium* to bind to the autophagosome membrane.

### Elongation and Lipid Transport Mediated by ATG2

Phagophore elongation requires lipid transport from the ER by the lipid transfer protein ATG2, which forms a complex with WIPI4 in mammalian cells (Bakula et al., 2017, 2018; Chowdhury et al., 2018; Maeda et al., 2019; Osawa et al., 2020; Sawa-Makarska et al., 2020) and Atg18 in yeast (Obara et al., 2008; Gómez-Sánchez et al., 2018; Kotani et al., 2018). A proteomic analysis suggests that WIPI4 forms a complex with AMPK and ULK1 under fed conditions and is released upon starvation to localize together with ATG2 at the phagophore (Bakula et al., 2017, 2018).

Functionally, both WIPI3 and WIPI4 are downstream of LC3 lipidation as their depletion in G316 cell lines leads to abnormal accumulation of other autophagic proteins such as WIPI1 and LC3 (Bakula et al., 2017). In addition, mice with double KO of WIPI3 and 4 show a more severe autophagy defect than single KO, suggesting that these two proteins act cooperatively in autophagy (Ji et al., 2020). In *Dictyostelium*, accumulation of Atg18 and Atg8 is also observed in cells lacking Wdr45l, suggesting that this protein is the functional counterpart of WIPI3 and 4 (Tornero-Écija et al., 2021). Thus, mammalian WIPI3 and 4, *Dictyostelium* Wdr45l and yeast Atg18 appear to be functionally equivalent, even though the yeast Atg18 belongs to the other phylogenetic group formed by WIPI1 and 2, *Dictyostelium* Atg18 and yeast Atg21. This is another clear example of the lack of correlation between sequence similarity and functional conservation.

### Fusion of Autophagosomes With Lysosomes

Defects in lipid transport during autophagosome formation resulting from lack of ATG2 or WIPI3/4 are expected to cause abnormal small phagophores to accumulate. However, a recent report showed that WIPI3/4 double KO mouse neuroblastome cells (N2a) can form autophagosomes that appear to be closed but are very small (Ji et al., 2021). The small size of these autophagosomes is overcome by overexpression of ATG2A, which is consistent with the involvement of ATG2/WIPI3/4 in lipid transfer and autophagosome membrane growth. Interestingly, WIPI3/4 appears to be required for a later stage of the autophagy pathway, as the fusion of these small autophagosomes with lysosomes is impaired in WIPI3/4 double KO (Ji et al., 2021). In the proposed model, WIPI3 or WIPI4 binds to EPG5, a protein required for the tethering of autophagosomes to lysosomes and the assembly of the SNARE complex necessary for fusion. As previously observed in the mouse model, autophagy defects are observed in WIPI3/4 double KO in N2a cells but not in single KOs of WIPI3 or WIPI4, again suggesting redundancy of function in this neural cell line.

### Non-autophagic Functions of WIPIs

The versatility and complexity of WIPI proteins are also evidenced by their function in pathways other than canonical autophagy. Indeed, WIPI1 (also known as WIPI49 for 49 KD) was initially characterized for its function in endosome trafficking. It localizes to the trans-Golgi and endosomes (in COS-7 cells) and siRNA-mediated KD in COS7 leads to impaired CI-MPR (mannose 6-P receptor) trafficking (Jeffries et al., 2004). Inactivation of WIPI1 leads to enlargement of endosomes and accumulation of endosomal membrane tubules. In addition, WIPI1 (but not the other WIPIs) has recently been shown to be required for transferrin receptor recycling to the plasma membrane in HK2 (human kidney) cells (De Leo et al., 2021). It is required for the formation of tubulo-vesicular endosomal transport carriers. The binding of the two phosphatidyl inositol binding sites to PtdIns3P or PI(3, 5)P2 appear to determine WIPI1 function in autophagy or endosomal trafficking. Interestingly, yeast Atg18 (formerly known as Svp1) is required for protein recycling from the vacuole to the Golgi and its inactivation leads to vacuole enlargement and accumulation of PtdIns(3, 5)P2 (Dove et al., 2004). PI(3, 5)P2 recruits Atg18 to the vacuole where it regulates retrograde transport of proteins from the vacuole to Golgi. Its function in autophagy is independent of binding to this lipid (Dove et al., 2004).

Human WIPI3 has been implicated in an alternative form of autophagy known as GOMED (Golgi-membrane-associated degradation) that is independent of the conjugation machinery and generates autophagic structures from Golgi membranes (Yamaguchi et al., 2020).

## Structural and Functional Features of WIPI Proteins: Interactions With Phosphoinositides and the Proteins Atg16 and Atg2

The crystal structure of Hsv2 from *Kluyveromyces lactis* revealed a characteristic structure shared by the entire WIPI protein family (Krick et al., 2012), corresponding to a seven-bladed β-propeller, each blade being formed by four antiparallel β-strands. This structure is reminiscent of a barrel-like structure and has a non-velcro closure, whereby the N-terminus is not part of the last C-terminal blade. Interaction with lipids and with proteins such as Atg2 and Atg16 involve different regions of the protein.

WIPIs bind to the membrane through two lipid-binding sites that require the conserved motif L/FRRG, which can be considered a signature of this family of proteins. Each R residue is in opposite directions, located in the two pockets (site1 and site 2) that are involved in phosphoinositide binding. Site 1 is located in blade 5 and is associated with one of the conserved R and the other site is located in blade 6 and is associated with the second R (Baskaran et al., 2012; Krick et al., 2012; Watanabe et al., 2012). *In vitro* analyses showed that WIPI1 (formerly known as WIPI49) binds preferentially to PtdIns3P and to a lesser extent to PtdIns5P and PtdIns(3, 5)P2 (Jeffries et al., 2004), while yeast Atg18 (Svp1) binds to both PtdIns3P and PI(3, 5)P2 (Dove et al., 2004; Strømhaug et al., 2004).

Site-directed mutagenesis analysis of the interaction between yeast Atg2 and Atg18, combined with yeast-two hybrid (Y2H) and co-immunoprecipitation studies, identified blade 2 and the region between blade 2 and 3 (loop 2) as the regions required for the interaction (Watanabe et al., 2012; Rieter et al., 2013). The interaction between mammalian ATG2 and WIPI4 proteins has been investigated by cross-linking mass spectrometry analysis and several lysine residues in blade 1 and blade 3 were found to cross-link to ATG2. In addition, alanine scanning mutagenesis located additional residues important for the interaction in loop 3 (the region between blade 3 and blade 4) (Zheng et al., 2017). These results suggest that the surface of interaction is extensive, which is consistent with results obtained from studies of the interaction between WIPI3 and the WIR (WIPI-interacting region), an ATG2A peptide that interacts with WIPI3. Structural analyses showed that the WIR peptide wraps around WIPI3 and binds to three sites in blades 1, 2, and 3 (Ren et al., 2020). Recently, a different site located on blade 7 has been proposed to be involved in the interaction between yeast Atg18 and Atg2 (Lei et al., 2021).

The interaction of the WIPI protein Atg21 with Atg16 has been characterized by the crystal structure of the yeast Atg16-Atg21 complex (using *K. lactis* Atg21 and *A. gossypii* Atg16). Three consecutive alpha-helical turns in Atg16 form a hydrophobic patch that inserts into a hydrophobic cleft of KIAtg21 between blades 2 and 3 (Munzel et al., 2020).

## WIPIs and Disease

Giving the important role of WIPI proteins in autophagy and other membrane-trafficking processes, it is not surprising that they play prominent roles in human health and disease. Indeed, mutations in any of the four WIPI proteins are associated with a variety of diseases affecting the nervous system (Table 1). Among these, mutations in WIPI4 have been extensively characterized concerning β-proppeler associated neurodegeneration (BPAN), a disease also referred to as SENDA (static encephalopathy of childhood with neurodegeneration in adulthood) or NBIA5 (neurodegeneration with brain iron accumulation-5). BPAN is an X-linked neurodegenerative disease characterized by developmental delay in early childhood that later progresses to other symptoms such as dystonia, parkinsonism and psychiatric disorders. BPAN is more common in females than in males, who tend to have more severe forms of the disease. The wide spectrum of disease phenotypes associated with WIPI4 mutations probably results from a combination of different factors, including mutation severity, somatic mosaicism and skewed X-chromosome inactivation in females.

**TABLE 1:** Missense mutations in WIPI proteins and associated diseases.

	Mutation	Disease*	References
WIPI1	R328Q	ANPH	Wang et al., 2019
	G313R	ANPH	Wang et al., 2019
	T418M	ANPH	Wang et al., 2019
	L406P	ANPH	Wang et al., 2019
WIPI2	V249M	IDDSSA	Jelani et al., 2019
WIPI3	R109Q	IDM	Najmabadi et al., 2011
WIPI4	N61K	BPAN	Haack et al., 2012
	D84G	BPAN	Long et al., 2015
	L98P	BPAN	Haack et al., 2012; Nishioka et al., 2015
	F100S	BPAN	Adang et al., 2020
	N202K	BPAN	Cong et al., 2021
	G205D	BPAN/DEE	Carvill et al., 2018; Chard et al., 2019
	A209D	BPAN	Tschentscher et al., 2015
	S210P	BPAN	Nishioka et al., 2015
	V66E	DEE	Khoury et al., 2019
	R134P	DEE	Chen et al., 2019
	G168E	DEE	Chen et al., 2019
	L160R	EOEE	Russo et al., 2019

*BPAN, β-propeller associated neurodegeneration;*

*DEE, Developmental and epileptic encephalopathy;*

*EOEE, Early onset epileptic encephalopathies;*

*ANPH, Anencephaly;*

*IDDSSA, Intellectual developmental disorder with short stature and variable skeletal anomalies;*

IDM, Intellectual disability and microcephaly.

*Nomenclature for WIPI4-related diseases was taken from Cong et al. (2021).

**FIGURE 4:**
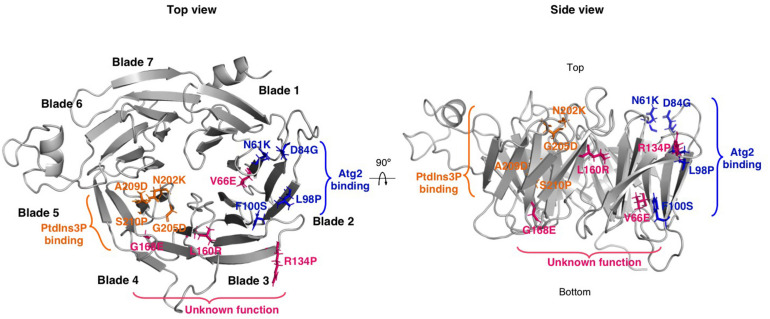
Localization of BPAN missense mutations in the 3D structure of WIPI4. WIPI4 structure was predicted using Robetta (http://robetta.bakerlab.org) based on the structure of WIPI3 (PDB: 6IYY). Top and side views of WIPI4 structure were generated with PyMOL software (The PyMOL Molecular Graphics System, Version 2.4.0 Schrödinger, LLC). Mutated residues localized in the PtdIns3P and ATG2 binding domains are shown as orange and blue sticks, respectively. Other mutated residues are shown as pink sticks.

Most of the mutations reported in WIPI4 are truncations, internal deletions and nonsense mutations that profoundly alter the structure of the protein, probably resulting in loss of function. However, some are missense mutations that result in the substitution of a single amino acid (Table 1). These types of mutations can be very informative, as they can point to key residues for the function of specific domains of the protein. This is the case of the BPAN mutations N202K, G205D, A209D, and S210P in WIPI4, which are all located in the PtdIns3P binding domain (Baskaran et al., 2012; Krick et al., 2012; Watanabe et al., 2012), and therefore could potentially alter the correct subcellular localization of the protein (Figure 4). In agreement with this hypothesis, the introduction of three of these mutations in the conserved residues of the WIPI4 homolog in *Dictyostelium*, Wdr45l, prevents its localization in autophagic structures. In addition, these mutant proteins do not complement the phenotypic defects of the KO mutant. These findings confirm the effect of these mutations on the localization of the protein during autophagy and the relevance of this localization *in vivo* (Tornero-Écija et al., 2021). Notably, a pathogenic mutation in WIPI2 (V249M) is also located in the PtdIns3P binding domain (Jelani et al., 2019), and is also likely to alter the localization of the protein. In the same line, the pathogenic mutations N61K, D84G, L98P, and F100S are all located in the Atg2 binding region (Figure 4) and the introduction of two of these mutations in the conserved residues of WIPI3 prevent binding to the ATG2A WIR sequence (Ren et al., 2020). Consistent with these findings, the introduction of three of these mutations in *Dictyostelium* Wdr45l inactivate the protein but do not alter the normal localization of the protein in autophagosomes (Tornero-Écija et al., 2021). Moreover, a pathogenic mutation in WIPI3 (R109Q) is also located in the ATG2 binding region and this mutation has been shown to impair ATG2A WIR binding (Ren et al., 2020). Finally, in addition to mutations in the PtdIns3P and ATG2A binding domains, other pathogenic missense mutations have been identified in WIPI4 (Figure 4). Notably, unlike the mutations described above, these mutations are not associated with BPAN but with other disorders such as developmental and epileptic encephalopathy (DEE) and early onset epileptic encephalopathy (EOEE) (Table 1).

The reason for the variety of clinical manifestations associated to WIPI4 mutations (see Cong et al., 2021 for a comprehensive review of the clinical symptoms) is not yet clear and could be due to the influence of genetic and environmental factors.

The severity of the phenotype of the Wdr45l KO in *Dictyostelium* is similar to that observed after expressing Wdr45l with BPAN mutations in the KO strain, suggesting that all these pathogenic mutations greatly affect protein function to the same extent as lack of the protein. This is consistent with the severity of the symptoms of BPAN patients, as no clear difference in severity is observed between missense mutations and those causing truncations or deletions of the protein. One exception is the mutation D84G that has been reported to cause milder symptoms in one patient (Stige et al., 2018). Notably, this mutation, as well as the pathogenic N61K mutation, is located in the ATG2 binding site. However, unlike N61K, D84G leads only to a partial defect in the interaction with ATG2, which could explain the unexpectedly mild phenotype (Bueno-Arribas et al., 2021).

## Insights From the *Dictyostelium* Model in WIPI4-Related Diseases

**FIGURE 5:**
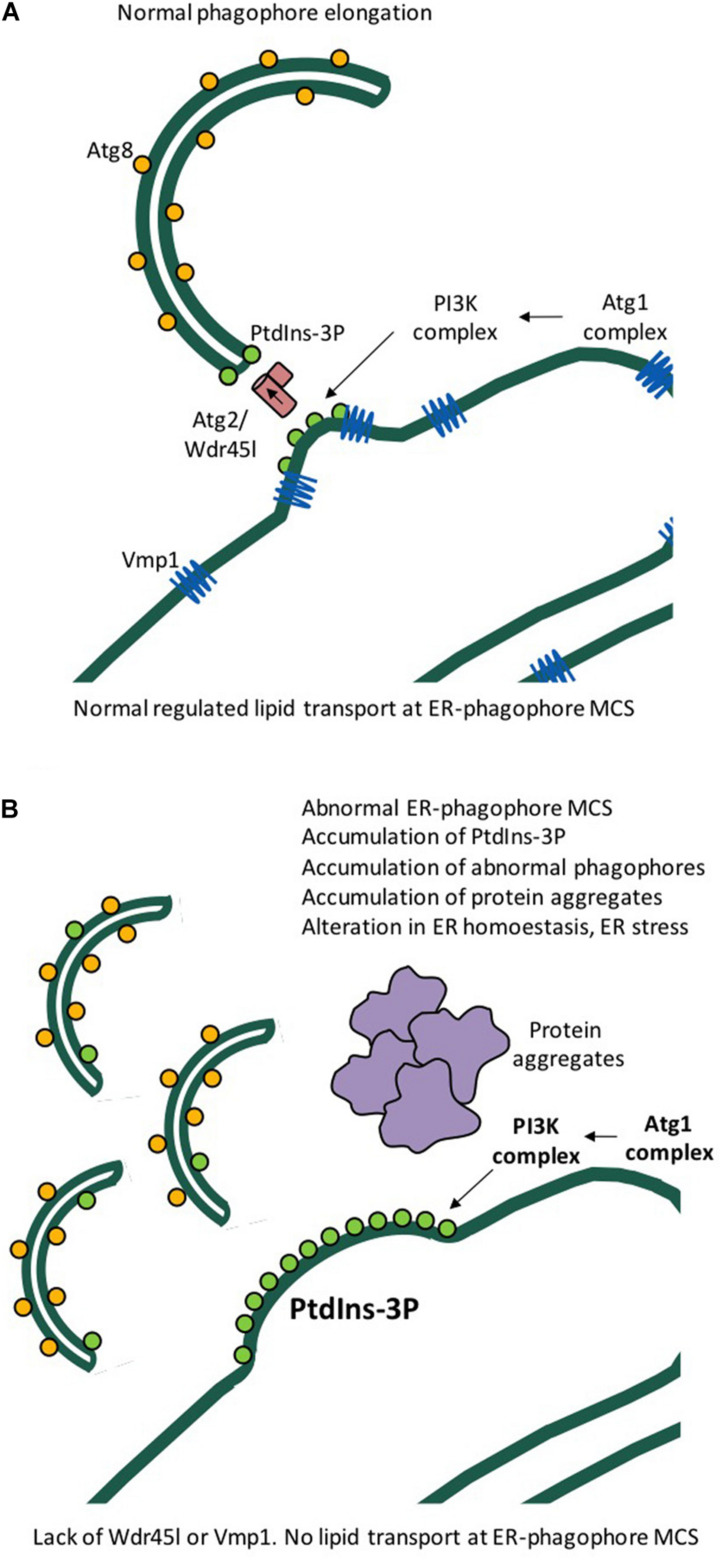
Model of phagophore expansion and the function of *Dictyostelium* Wdr45l and Vmp1. **(A)** Lipid transport and phagophore elongation from the ER occur at membrane contact sites (ER-phagophore MCS) and require Atg2, Wdr45l, and Vmp1. **(B)** Cells deficient in Vmp1 or Wdr45l have similar phenotypes, including the accumulation of PtdIns3P, abnormal autophagic structures, and formation of protein aggregates. Abnormal accumulation of PtdIns3P leads to chronic ER stress and cell growth arrest. These defects are rescued by inhibition of the upstream signaling protein Atg1.

KO of Wdr45l, the functional homolog of human WIPI4 in *Dictyostelium*, impairs growth and development in this organism, a phenotype that is remarkably similar to the KO of Vmp1 (Tornero-Écija et al., 2021). Vmp1 is an essential protein in autophagy that is conserved from *Dictyostelium* to humans but is absent in yeast (Calvo-Garrido et al., 2008). Mammalian VMP1 plays an essential role in organellar communication by regulating the function of membrane contact sites (MCS) between the ER and other organelles, including mitochondria, endosomes, peroxisomes and also the phagophore membrane during elongation (Tábara and Escalante, 2016; Zhao et al., 2017). Consistent with this variety of localizations, *Dictyostelium* Vmp1 KO exhibits pleiotropic defects affecting organellar morphology, protein sorting, and autophagy (Calvo-Garrido et al., 2008; Calvo-Garrido and Escalante, 2010). It has been recently proposed that the scramblase activity of VMP1 is essential for lipid trafficking between the ER membrane and the phagophore (Ghanbarpour et al., 2021; Li et al., 2021). The current model predicts that the lack of any of the components involved in lipid trafficking, such as the WIPI-ATG2 complex and the VMP1 and ATG9 scramblases, may lead to similar or related phenotypes. This appears to be the case for *Dictyostelium* Vmp1 and Wdr45l, the absence of which leads to similar phenotypes, including a block in autophagy downstream of Atg8 lipidation and accumulation of PtdIns3P at the autophagosome assembly site. This accumulation appears to be responsible for additional phenotypes, including, interestingly, chronic activation of the endoplasmic reticulum (ER) stress response (also known as UPR, unfolded protein response) (Figure 5). The UPR is a complex pathway triggered by the accumulation of unfolded proteins that regulates many aspects of cellular physiology and is closely related to neurodegenerative diseases (van Ziel and Scheper, 2020). In *Dictyostelium*, the UPR regulates a gene expression program that inhibits cell growth and global protein synthesis, while activating specific genes encoding proteins that will increase the folding and degradation capacity of cells (Domínguez-Martín et al., 2018a,b). Notably, mutation of the key upstream regulator Atg1 suppresses growth and UPR activation phenotypes by preventing PtdIns3P formation (Tornero-Écija et al., 2021). The possible role of the UPR pathway in BPAN is reinforced by the observation of WIPI4-deficient neurons from a KO mouse showing accumulation of misfolded proteins and activation of the ER-stress response (Wan et al., 2019). If a similar relationship between UPR and abnormal autophagy-associated PtdIns3P occurs in human cells, attenuation of PtdIns3P signaling could be a therapeutic target (Figure 5). The *Dictyostelium* model also suggests that mutations in any other component of the lipid transport machinery such as VMP1 and ATG2 could also lead to neurological diseases similar to BPAN.

## Open Questions and Future Directions

The function of WIPI proteins is complex and not yet clearly established. Although they are phosphatidylinositol effectors that recognize at least PtdIns3P and PtdIns(3, 5)P2, their interaction with membranes is dictated not only by the presence of the signaling lipid. For example, *Dictyostelium* Atg18 and human WIPI1 are used as autophagic markers because of their localization at autophagic structures, as they seem to preferentially recognize autophagy-associated PtdIns3P despite the abundant presence of PtdIns3P in other cellular locations (Domínguez-Martín et al., 2017; Klionsky et al., 2021). Therefore, it is important to further investigate where and how the different WIPIs localize, and what determinants regulate their localizations. WIPIs interact with many other proteins and it is often unclear whether they act simply to recruit other proteins to particular locations or have a regulatory role as well (Bakula et al., 2017). An example of this second function has been described for WIPI2, which not only recruits but also activates the lipidation machinery (Fracchiolla et al., 2020).

WIPIs appear to have both specific and redundant functions, which further complicates their characterization and the study of their role in disease. Although autophagy appears to play a prominent role in many neurodegenerative diseases, it is unclear whether the cytopathological alterations in WIPI-related diseases are caused by defects in other autophagy-independent processes, such as the ER-stress response pathway. Many basic questions remain to be answered before effective knowledge-based treatments for these incurable diseases are feasible. In this complex scenario, the use of simple models such as yeast and *Dictyostelium* may lead to new avenues of discovery and understanding the function of WIPI proteins in the cell.

## Author Contributions

OV: bibliographic study and manuscript writing. LA-E, MB-A, and AT-É: revision of the manuscript and figure preparation. M-ÁN: revision of manuscript. RE: coordination, bibliographic study, and manuscript writing. All authors contributed to the article and approved the submitted version.

## Conflict of Interest

The authors declare that the research was conducted in the absence of any commercial or financial relationships that could be construed as a potential conflict of interest.

## Publisher’s Note

All claims expressed in this article are solely those of the authors and do not necessarily represent those of their affiliated organizations, or those of the publisher, the editors and the reviewers. Any product that may be evaluated in this article, or claim that may be made by its manufacturer, is not guaranteed or endorsed by the publisher.
